# Diagnostic reference levels and median doses for common clinical indications of CT: findings from an international registry

**DOI:** 10.1007/s00330-021-08266-1

**Published:** 2021-10-13

**Authors:** Denise Bos, Sophronia Yu, Jason Luong, Philip Chu, Yifei Wang, Andrew J. Einstein, Jay Starkey, Bradley N. Delman, Phuong-Anh T. Duong, Marco Das, Sebastian Schindera, Allen R. Goode, Fiona MacLeod, Axel Wetter, Rebecca Neill, Ryan K. Lee, Jodi Roehm, James A. Seibert, Luisa F. Cervantes, Nima Kasraie, Pavlina Pike, Anokh Pahwa, Cécile R. L. P. N. Jeukens, Rebecca Smith-Bindman

**Affiliations:** 1 Institute of Diagnostic and Interventional Radiology and Neuroradiology, University Hospital Essen, Hufelandstrasse 55, 45147 Essen, Germany; 2 Department of Epidemiology and Biostatistics, University of California San Francisco, 550 16th Street, San Francisco, CA 94158 USA; 3 Department of Medicine, Seymour, Paul, and Gloria Division of Cardiology, and Department of Radiology, Columbia University Irving Medical Center, 622 West 168th Street, PH 10-203B, New York, NY 10032 USA; 4 Radiology St Luke’s International Hospital, 9-1 Akashicho, Chuo City, Tokyo 104-8560 Japan; 5 Department of Diagnostic, Molecular and Interventional Radiology, Icahn School of Medicine at Mount Sinai, One Gustave L. Levy Place, New York, NY 10029-6574 USA; 6 Department of Radiology and Imaging Sciences, University of Utah, 50 N Medical Dr, Salt Lake City, UT 84132 USA; 7 Department of diagnostic and interventional Radiology, Helios Hospital Duisburg, An der Abtei 7-11, 47166 Duisburg, Germany; 8 Institute of Radiology, Kantonsspital Aarau AG, Tellstrasse 25, 5001 Aarau, Switzerland; 9 Department of Radiology and Medical Imaging, UVA Health, 1215 Lee St, Charlottesville, VA 22908 USA; 10 Radiology, John Radcliffe Hospital, Headley Way, Headington, OX3 9DU Oxford UK; 11 Department of Radiology and Imaging Sciences, Emory University, 1365 Clifton Road NE, AT 631-A, Atlanta, GA 30322 USA; 12 Department of Radiology, Einstein Healthcare Network, 5501 Old York Road, Philadelphia, PA 19141 USA; 13 RAYUS Radiology, 5775 Wayzata Blvd., St. Louis Park, MN 55416 USA; 14 Department of Radiology, UC Davis Health, 4860 Y Street, Sacramento, CA 95817 USA; 15 Department of Radiology, Nicklaus Children’s Hospital, 3100 SW 62nd Avenue, Miami, FL 33155 USA; 16 Radiology, UT Southwestern Medical Center, 1801 Inwood Rd, Dallas, TX 75235 USA; 17 Huntsville Hospital, 101 Sivley Rd SW, Huntsville, AL 35801 USA; 18 Department of Radiological Sciences, Olive View - UCLA Medical Center, 14445 Olive View Dr, Sylmar, CA 91342 USA; 19 Department of Radiology and Nuclear Medicine, Maastricht University Medical Centre +, P. Debyelaan 25, 6229 HX Maastricht, Netherlands; 20 Philip R Lee Institute for Health Policy Studies, University of California San Francisco, 3333 California St, San Francisco, CA 94118 USA

**Keywords:** Tomography, X-ray computed, Adult, Diagnostic reference levels, United States, Registries

## Abstract

**Ob jectives:**

The European Society of Radiology identified 10 common indications for computed tomography (CT) as part of the European Study on Clinical Diagnostic Reference Levels (DRLs, EUCLID), to help standardize radiation doses. The objective of this study is to generate DRLs and median doses for these indications using data from the UCSF CT International Dose Registry.

**Methods:**

Standardized data on 3.7 million CTs in adults were collected between 2016 and 2019 from 161 institutions across seven countries (United States of America (US), Switzerland, Netherlands, Germany, UK, Israel, Japan). DRLs (75th percentile) and median doses for volumetric CT-dose index (CTDI_vol_) and dose-length product (DLP) were assessed for each EUCLID category (chronic sinusitis, stroke, cervical spine trauma, coronary calcium scoring, lung cancer, pulmonary embolism, coronary CT angiography, hepatocellular carcinoma (HCC), colic/abdominal pain, appendicitis), and US radiation doses were compared with European.

**Results:**

The number of CT scans within EUCLID categories ranged from 8,933 (HCC) to over 1.2 million (stroke). There was greater variation in dose between categories than within categories (*p *< .001), and doses were significantly different between categories within anatomic areas. DRLs and median doses were assessed for all categories. DRLs were higher in the US for 9 of the 10 indications (except chronic sinusitis) than in Europe but with a significantly higher sample size in the US.

**Conclusions:**

DRLs for CTDI_vol_ and DLP for EUCLID clinical indications from diverse organizations were established and can contribute to dose optimization. These values were usually significantly higher in the US than in Europe.

**Key Points:**

• *Registry data were used to create benchmarks for 10 common indications for CT identified by the European Society of Radiology.*

• *Observed US radiation doses were higher than European for 9 of 10 indications (except chronic sinusitis).*

• *The presented diagnostic reference levels and median doses highlight potentially unnecessary variation in radiation dose.*

**Supplementary Information:**

The online version contains supplementary material available at 10.1007/s00330-021-08266-1.

## Introduction

Radiation doses for computed tomography (CT) are highly variable across patients, institutions, and countries [1–5]. Because of the rising use of CT [6–8], the carcinogenic risk of ionizing radiation [9–14], and the relatively high radiation doses associated with CT, greater standardization is needed across institutions and countries for CT dose [4, 15]. Variation in radiation doses at CT is primarily attributable to how the scans are conducted and the setting of technical parameters, rather than patient, institutional, or machine characteristics, all of which have a smaller contribution to variation in observed doses [4]. Thus, dose optimization can be achieved across most machine types through optimization of technical parameters. The establishment of diagnostic reference levels (DRLs) was first implemented by the International Commission on Radiological Protection (ICRP) in 1996 to aid in optimization of medical radiation exposures [16]. The DRLs were identified as doses that should not be exceeded on a patient of routine size unless there was a particular need to do so. Some organizations require the documentation of medical necessity if these values are exceeded. The European Council Directive 2013/59/EURATOM requires the establishment, regular review, and use of DRLs for member states [15]. The US National Council on Radiation Protection and Measurements (NCRP) implemented an additional concept of achievable doses (ADs) in 1999 reflecting the median. These doses were identified as doses that should help improve further dose optimization and encourage faculties to achieve lower doses than the DRL [17, 18].

DRLs and achievable doses are commonly used in clinical practice to guide radiology facilities and practitioners in efforts to optimize CT doses. Most commonly, the DRL is set at the 75th percentile of the dose distribution and the achievable dose at the 50th percentile for a geographical area [2, 17–19]. These are often created broadly within anatomic areas, and yet even within anatomical regions, different clinical indications have different image quality requirements.

The European study on clinical diagnostic reference levels for X-ray medical imaging (EUCLID), funded by the European Commission and led by the European Society of Radiology, has established clinical indication-based DRLs for ten common clinical indications to provide better guidance to radiologists and technologists. In this large-scale survey, dose data of 4299 adult patients with standard size were collected across 14 European countries via an online survey [20, 21]. Since the EUCLID categories provide a useful approach for setting dose targets and thresholds, we would expect to see greater variation between the EUCLID categories than within the EUCLID categories because of the different image quality requirements of different CT indications. In this study, we used data from the University of California San Francisco (UCSF) CT International Dose Registry to describe DRLs and median doses for EUCLID clinical indications and compare radiation doses between facilities of the United States of America (US) and Europe (EU).

## Materials and methods

### UCSF CT International Dose Registry

Details regarding the UCSF CT International Dose Registry have been previously described [4]. In short, the registry includes imaging data from 161 imaging facilities and hospitals associated with 27 healthcare institutions from seven countries (United States of America (US), Switzerland, Netherlands, Germany, UK, Israel, and Japan) that use Radimetrics dose management software (Radimetrics^TM^ Enterprise Platform, Bayer AG, Leverkusen, Germany). Radimetrics was selected as the only one of several available dose management software because it was already being used by several hospitals internationally at the time the dose registry was initiated. Due to the need for secure data transfer, only one dose management software was used as data source. All customers of Radimetrics were invited to participate in the registry, and institutions who elected to participate and were able to meet the logistical requirements such as establishing data connections and receiving institutional approval to share data are included. At each imaging facility, imaging data are assembled on a local server, stripped of patient identifying information and then transferred to the registry [4]. The UCSF Committee on Human Research and the institutional review boards of the collaborating institutions approved the study, waived informed consent, or relied on the UCSF approval.

### Study population

Data are included for diagnostic CT examinations performed in adult patients ages 18 years and older between 1 January 2016 and 31 December 2019. All patient sizes were included. Exams for research, surgical or interventional procedures, combined with positron emission tomography (i.e., PET-CT), or single photon emission tomography (i.e., SPECT-CT), and done for radiation oncology guidance were excluded.

### Indication for CT examination

The indication for each CT examination was determined by applying natural language processing techniques to the study description and protocol name included in the Digital Imaging and Communications in Medicine (DICOM) metadata. This included searching for explicit terms and classifying exams using the most specific and highest dose category. In the EUCLID study, ten common clinical indications for undergoing CT were identified [21]. These EUCLID clinical indications or categories include (1) sinus to asses for sinusitis and polyps; (2) stroke, which we aligned with a routine brain examination, and imaging of the brain to assess for hemorrhage; (3) cervical spine for trauma; (4) coronary calcium scoring; (5) lung cancer, which we aligned with routine chest examinations, as well as cancer diagnosis and staging, and not screening; (6) pulmonary embolism; (7) coronary angiography; (8) hepatocellular carcinoma (HCC), suspected or evaluation of known; (9) colic/abdominal pain, which we aligned with imaging for suspected kidney stones; and (10) abdominal pain, suspected appendicitis, which we aligned with routine abdomen. EUCLID category of “stroke” most closely aligns with a routine head CT performed to exclude hemorrhage, so brain perfusion scans and cerebrovascular CT angiograms were excluded.

### Dose metrics

Results are provided for complete CT examinations including all scans performed as part of the examination (e.g., a complete examination would include all imaging acquisitions and might include one scan with and one without intravenous contrast). Bolus scans were identified and the dose was included in the total exam dose, but these scans were not considered when determining examination phase. Results are reported for volumetric CT-dose index (CTDI_vol_) reflecting the average radiation exposure per section and the dose-length product (DLP) reflecting the total radiation output for the examination. Patient diameter was defined as the average of the water equivalent diameter from each CT acquisition over the entire imaging range and used to adjust radiation doses by size [4, 22].

### Statistical analysis

For each clinical indication, the median dose was defined as the 50th percentile in dose distribution and the DRL was defined as the 75th percentile in dose distribution. The 95% confidence intervals (CI) of the DRLs and median doses were calculated by bootstrapping using random sampling with replacement [23].

Analysis of variance was used to determine if there was greater variation between categories, as compared to within categories. The distribution of DRLs and median doses were assessed for the United States (US) and Europe (EU) after adjusting for patient size and age. For the international comparison, only US and European CT exams were investigated further because the registry includes sufficient sample sizes for each EUCLID category; sample sizes for facilities in Japan and Israel were insufficient. The adjusted doses were estimated via log-linear regression estimating the dose for patients with average age and size. Bootstrapping within each country was used to determine if the DRLs and median doses varied by country when stratified by anatomic area. The relative DRLs and relative median doses (and 95% CI) between the US and Europe were calculated. Analysis was done using R version 3.6.3.

## Results

A total of 3,718,217 CT scans performed between January 1, 2016, and December 31, 2019, across the 10 EUCLID clinical indications are included (Table 1). The number of CT scans for each indication ranged from 8,933 for hepatocellular carcinoma to over 1.2 million for stroke. The examinations were performed on 383 CT scanners across 74 machine models from the four largest CT manufacturers. Most of the 161 participating facilities contributed to most of the clinical indications with some notable exceptions, e.g., coronary calcium scoring was recorded at only 73 facilities, coronary angiography at 66 facilities, and HCC at 19 facilities. Overall 84 % of all CT examinations were performed at the US facilities (*n *= 141) and 8 % at the European facilities (*n *= 11).

**Table 1:** The number of CT scans included for each EUCLID category, and the number of CT models, individual CT machines, and imaging facilities that contributed to the category.

Body region	EUCLID category	*n*	CT models	CT machines	Facilities
All combined		3,718,217	74	383	161
Head	Chronic sinusitis^a^	72,838	64	317	144
	Stroke^b^	1,270,008	74	372	161
Neck	Cervical spine trauma	250,781	64	319	137
Chest	Coronary calcium scoring	18,828	30	135	73
	Lung cancer^c^	639,884	74	368	159
	Pulmonary embolism	143,736	55	230	98
	Coronary CT angiography	32,550	27	135	66
Abdomen	Hepatocellular carcinoma	8,933	15	42	19
	Colic/abdominal pain	107,816	55	277	105
	Appendicitis^d^	1,172,843	74	374	161

^a^Mapped to sinus CT in the dose registry

^b^Mapped to routine head CT in the dose registry

^c^Mapped to routine chest CT and imaging for known or suspected lung cancer in the dose registry

^d^Mapped to routine abdomen CT in the dose registry

### DRLs and median doses

**Fig. 1:**
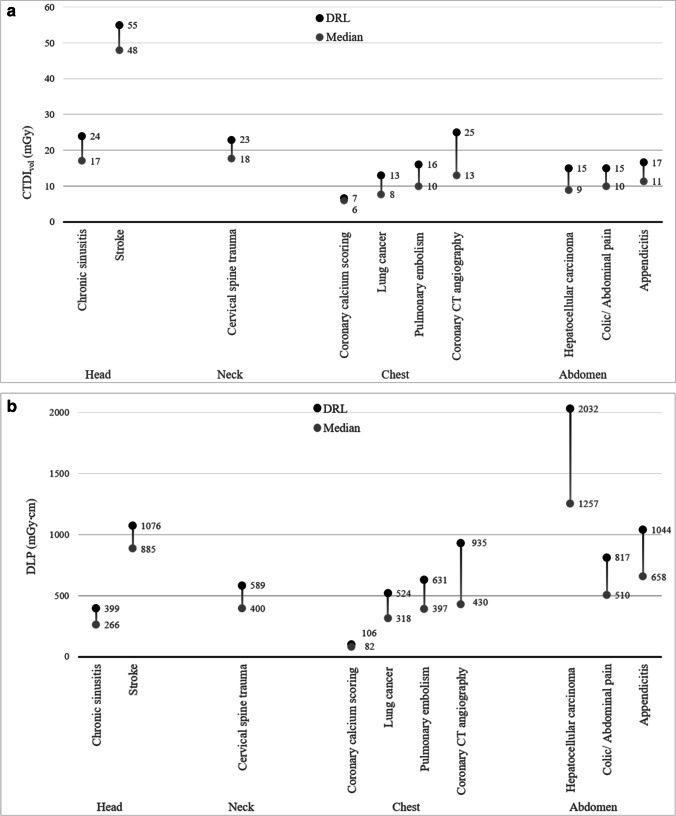
Distribution in dose metrics for CTDI_vol_ (**a**) and DLP (**b**) for each EUCLID category, showing median doses (50th, gray) and diagnostic reference levels (DRLs) (75th, black). **a** Distribution of CTDI_vol_ (in mGy) for each EUCLID indication. **b** Distribution of DLP (in mGy cm) for each EUCLID indication.

There was greater variation for CTDI_vol_ and DLP between categories, as compared to within categories (*p* < .001) (Table 2 and Fig. 1). Within each anatomic area, the DRLs and median doses were significantly different across the EUCLID categories (Table 2, Fig. 1, *p* < .05). For example, in the chest the median doses for CTDI_vol_ increased from 5.7 mGy for coronary calcium scoring to 8.0 mGy for lung cancer, to 10.4 mGy for pulmonary embolism, to 13.3 mGy for coronary CT angiography, reflecting more than a 2-fold difference among the four chest categories (Table 2, Fig. 1a). The corresponding DRLs for CTDI_vol_ varied more than 3-fold ranging from 7.0 mGy for coronary calcium scoring to 25.4 mGy for coronary CT angiography. Even larger differences across these chest categories were observed for the DRLs and median values of DLP (Table 2 and Fig. 1b). For chest imaging, a 9-fold difference in the DRLs for DLP was observed, ranging from 106 mGy cm [95% CI 106, 107] for coronary calcium scoring to 935 mGy cm [95% CI 920, 949] for coronary CT angiography. The CI were narrow due to large sample size. Within each anatomic area, the differences between the EUCLID categories for median doses and DRLs were significantly different (all *p*-values < .05). The corresponding values adjusted for patient size changed relatively little (supplement 1).

**Table 2:** Diagnostic reference levels (DRLs) and median doses (and 95% CI) for CTDI_vol_ and DLP by EUCLID category. *p*-values shown are for differences between EUCLID categories within anatomic areas.

			CTDI_vol_ (mGy)				DLP (mGy cm)			
Body Region	EUCLID category	Percentage of multiphase exams*	Median [95% CI]	*p*-value	DRL [95% CI]	*p*-value	Median [95% CI]	*p*-value	DRL [95% CI]	*p*-value
Head	Chronic sinusitis	5%	17.1 [17.0; 17.1]	< 0.05	24.3 [23.9; 24.9]	< 0.05	266 [264; 268]	< 0.05	399 [397; 401]	< 0.05
	Stroke	12%	48.2 [48.2; 48.2]		55.2 [55.2; 55.2]		885 [885; 886]		1076 [1076; 1076]	
Neck	Cervical spine trauma	14%	17.6 [17.5; 17.6]		23.0 [23.0; 23.1]		400 [399; 401]		589 [587; 591]	
Chest	Coronary calcium scoring	6%	5.7 [5.7; 5.7]	< 0.05	7.0 [7.0; 7.0]	< 0.05	82 [82; 83]	< 0.05	106 [106; 107]	< 0.05
	Lung cancer	15%	8.0 [8.0; 8.0]		12.6 [12.6; 12.6]		318 [317; 319]		524 [523; 525]	
	Pulmonary embolism	23%	10.4 [10.3; 10.4]		15.7 [15.6; 15.8]		397 [395; 398]		631 [628; 634]	
	Coronary CT angiography	65%	13.3 [13.2; 13.5]		25.4 [25.0; 25.8]		430 [423; 436]		935 [920; 949]	
Abdomen	Hepatocellular carcinoma	97%	9.1 [8.9; 9.3]	< 0.05	14.6 [14.3; 14.9]	< 0.05	1257 [1236; 1281]	< 0.05	2032 [1992; 2070]	< 0.05
	Colic/abdominal pain	7%	9.8 [9.8; 9.9]		15.4 [15.3; 15.4]		510 [507; 513]		817 [812; 822]	
	Appendicitis	26%	11.4 [11.4; 11.5]		16.8 [16.8; 16.9]		658 [657; 659]		1044 [1042; 1046]	

^*^Multiphase exams require two or more phases

### Comparison between US and Europe

**Fig. 2:**
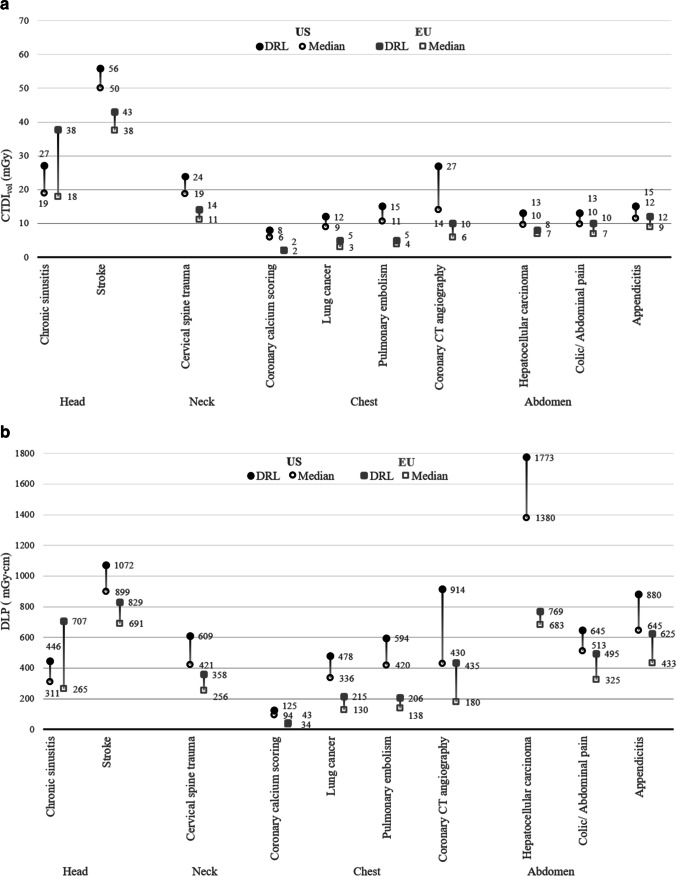
Distribution of dose metrics (**a** CTDI_vol_, **b** DLP) for each EUCLID category with comparison between the United States (US) and Europe (EU), showing median doses (50th percentile, black open circle for US and gray open box for EU), and diagnostic reference levels (DRLs) (75th percentile, black circle for US and gray box for EU). Doses were adjusted for patient size and age. **a** Comparison of DRLs and median doses for CTDI_vol_ (in mGy) between US and Europe for each EUCLID indication. **b** Comparison of DRLs and median doses for DLP (in mGy cm) between US and Europe for each EUCLID indication.

For most (9 of the 10) EUCLID categories, after adjusting for patient size and age, the DRLs and median doses were significantly higher in the US (all *p* < .05, Tables 3 and 4, Fig. 2a and b). The largest differences were seen for chest CT. Using CTDI_vol_, pulmonary embolism had an approximately 3-fold higher dose in the US (relative median dose 2.96 [95% CI 2.92, 3.00], relative DRL 2.73 [95% CI 2.70, 2.76]), and coronary calcium scoring had an approximately 4-fold higher dose in the US (relative median dose 3.92 [95% CI 3.85, 4.00], relative DRL 4.25 [95% CI 4.15, 4.38]). Lung cancer had an approximately 2-fold higher dose in the US (relative median dose 2.51 [95% CI 2.50, 2.52], relative DRL 2.22 [95% CI 2.20, 2.24]). Coronary CT angiography had a 2-fold higher dose in the US. In the US for other categories, the doses were modestly higher, i.e., approximately 22 to 77% higher (range in the relative median dose 1.31 to 1.67 and relative DRLs 1.22 to 1.77). US doses were comparable or lower only for chronic sinusitis (relative median dose 1.06 [95% CI 1.05, 1.06], relative DRL 0.72 [95% CI 0.71, 0.72]). Similar results were seen for DLP (Table 4 and Fig. 2b). For example, pulmonary embolism had an approximately 3-fold higher dose in the US (relative median dose 3.05 [95% CI 2.99, 3.10], relative DRL 2.89 [95% CI 2.84, 2.94]).

**Table 3:** Observed diagnostic reference levels (DRLs) and median doses for CTDI_vol_ (in mGy) in the United States (US) and Europe (EU), and relative DRLs and median doses in the US compared with Europe (and 95% CI) for EUCLID indications. Doses were adjusted for patient size and age.

Body region	EUCLID category	US		EU		Relative median US/EU [95% CI]		Relative DRL US/EU [95% CI]	
		Median	DRL (75th)	Median	DRL (75th)				
Head	Chronic sinusitis	18.6	26.9	17.6	37.5	1.06	[1,05; 1.06]	0.72	[0.71; 0.72]
	Stroke	49.7	56.2	37.8	42.6	1.32	[1,32; 1.32]	1.32	[1.32; 1.32]
Neck	Cervical spine trauma	18.8	24.1	11.3	13.6	1.67	[1,66; 1.68]	1.77	[1.76; 1.79]
Chest	Coronary calcium scoring	6.1	8.0	1.6	1.9	3.92	[3,85; 4.00]	4.25	[4.15; 4.38]
	Lung cancer	8.8	11.9	3.5	5.3	2.51	[2,50; 2.52]	2.22	[2.20; 2.24]
	Pulmonary embolism	11.1	14.9	3.7	5.5	2.96	[2,92; 3.00]	2.73	[2.70; 2.76]
	Coronary CT angiography	13.7	26.5	5.5	9.6	2.49	[2,41; 2.62]	2.76	[2.66; 2.85]
Abdomen	Hepatocellular carcinoma	9.8	12.5	6.9	7.7	1.42	[1,40; 1.45]	1.62	[1.58; 1.66]
	Colic/abdominal pain	10.0	12.6	6.9	9.5	1.44	[1,43; 1.46]	1.32	[1.29; 1.35]
	Appendicitis	11.6	14.5	8.9	11.9	1.31	[1,30; 1.31]	1.22	[1.22; 1.23]

**Table 4:** Observed diagnostic reference levels (DRLs) and median doses for DLP (in mGy cm) in the United States (US) and Europe (EU), and relative DRLs and median doses (and 95% CI) in the US compared with Europe for EUCLID indications. Doses were adjusted for patient size and age.

Body region	EUCLID category	US		EU		Relative median US/EU [95% CI]		Relative DRL US/EU [95% CI]	
		Median	DRL (75th)	Median	DRL (75th)				
Head	Chronic sinusitis	311	446	265	707	1.17	[1.15; 1.20]	0.63	[0.63; 0.64]
	Stroke	899	1,072	691	829	1.30	[1.30; 1.30]	1.29	[1.29; 1.30]
Neck	Cervical spine trauma	421	609	256	358	1.65	[1.63; 1.66]	1.70	[1.67; 1.73]
Chest	Coronary calcium scoring	94	125	34	43	2.72	[2.65; 2.77]	2.92	[2.85; 3.01]
	Lung cancer	336	478	130	215	2.59	[2.58; 2.60]	2.23	[2.20; 2.25]
	Pulmonary embolism	420	594	138	206	3.05	[2.99; 3.10]	2.89	[2.84; 2.94]
	Coronary CT angiography	430	914	180	435	2.39	[2.25; 2.55]	2.10	[2.01; 2.18]
Abdomen	Hepatocellular carcinoma	1,380	1,773	683	769	2.02	[1.97; 2.06]	2.30	[2.23; 2.35]
	Colic/abdominal pain	513	645	325	495	1.58	[1.56; 1.60]	1.30	[1.28; 1.33]
	Appendicitis	645	880	433	625	1.49	[1.48; 1.50]	1.41	[1.40; 1.41]

The use of multiphase scanning was similar for some indications between the US and Europe. For example, 99% of examinations performed for hepatocellular carcinoma use multiphase scanning in both the US and Europe. However, for other indications there were substantial differences between US and Europe in multiphase scanning: coronary CT angiography (66% vs 46%), colic/abdominal pain (6% vs 29%), and stroke (11% vs 18%).

### Comparison with published literature

**Fig. 3:**
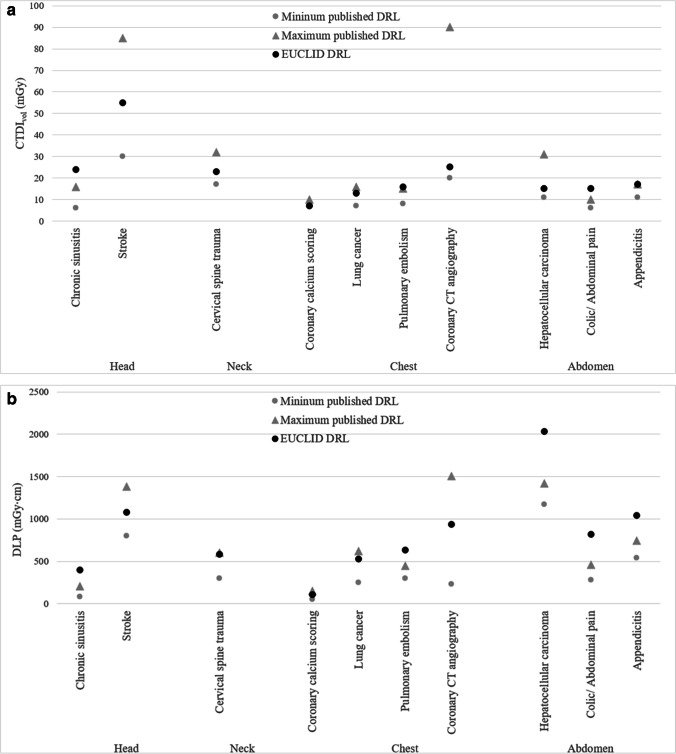
Comparison between published diagnostic reference levels (DRLs) (**a** CTDI_vol_ and **b** DLP) and observed DRLs in this report for each EUCLID indication. The maximum published value is shown with a triangle, the minimum published value with a circle (each in gray). The observed values are shown in black circles (EUCLID). **a** Comparison between published and observed DRLs for CTDI_vol_ of each EUCLID indication. **b** Comparison between published and observed DRLs for DLP of each EUCLID indication.

Most publications describing benchmark doses focus on the DRLs (75th percentile). The DRLs for DLP of the EUCLID indications of stroke, cervical spine trauma, coronary calcium scoring, coronary CT angiography, and lung cancer were within the range of values reported in the literature (Fig. 3b, supplement 2). The reported values are based on the literature review from the EUCID study of the European Society of Radiology and additional database research in PubMed [24]. For several EUCLID indications, including chronic sinusitis, pulmonary embolism, and the abdominal imaging categories (colic/abdominal pain, appendicitis, and HCC), the observed DRLs for DLP were higher than reported in the literature. For CTDI_vol_ the observed values were within published values for most EUCLID indications (Fig. 3a, supplement 2). Only DRLs for chronic sinusitis, pulmonary embolism, and colic/abdominal pain were higher.

Adjusted for patient age and size, US DRLs exceeded published DRLs for 6 of 10 indications (chronic sinusitis, cervical spine trauma, pulmonary embolism, all abdominal indications) in terms of DLP and twice in terms of CTDI_vol_ (chronic sinusitis and colic/abdominal pain). European DRLs exceeded published DRLs for DLP only for sinusitis and colic/abdominal pain, and DRLs for CTDI_vol_ exceeded only for sinusitis. For 4 of 10 indications for DLP (coronary calcium scoring, lung cancer, pulmonary embolism, HCC) and 6 of 10 indications for CTDI_vol_ (cervical spine trauma, all chest indications, HCC), European doses were lower than published DRLs.

Compared with recently published results from the large-scale, survey-based EUCLID study on standard-sized patients, most DRLs from the UCSF CT International Dose Registry, which includes all patient sizes, were higher than DRLs from the EUCLID study, except for stroke and lung cancer [21].

## Discussion

The DRLs and median doses for EUCLID indications included in this report can be used as a starting point for dose optimization. Because the observed large differences across the EUCLID categories greatly exceeded the dose differences within categories, our results support the use of the EUCLID clinical indication-specific CT protocols, rather than combining all indications within anatomic areas.

The advantage of these data is their large size, inclusion of all examinations as opposed to sampled examination, inclusion of a large number of different machine makes and models, and representation of data from 161 imaging facilities who participate in the UCSF CT International Dose Registry. These data primarily reflect US practice, and all were assembled from facilities that invested in dose management software, which should also be mentioned as limitations of the study. Because fewer European countries are represented, it is impossible to know if these reflect European practice more broadly or just the practice of a small number of imaging facilities in the small number of included countries.

The European Union has asked that member nations locally set DRLs for CT [15]. This is based on the presumption that doses vary by machine manufacturer and model and therefore must be set in a way that takes into consideration the type of equipment. We have shown previously that within our registry, machine make and model are relatively small contributors to dose variability, whereas how machines are used and the choice of technical parameters are the largest contributors of dose variability [4]. Thus, these benchmarks should be relevant for most imaging centers.

The large differences in doses between the US and Europe persisted after accounting for patient size and age. Inter-country variability results from the way the machines are used, the choice of technical parameters, and the decisions that clinical staff make to alter dose levels and hence contribute to dose variation [4]. The reasons for these differences are likely multifactorial including greater awareness of the need to optimize dose in Europe, which is reflected by the creation of EUCLID categories in the first place. Differences in the usage of multiphase scanning, e.g., coronary CT angiography, could reflect different practice cultures between Europe and the US. Reducing the number of acquired series is the easiest strategy to reduce radiation dose.

We present both DRLs and median doses for ten common clinical indications for CT, which represent the current status quo of the used radiation doses from the participating facilities. ICRP Publication 135 describes that DRLs are not intended to be used as a trigger or alarm level for individual patients and that DRLs are not limits. They can be used as benchmarks for further investigations when a representative sample of examinations exceed the local, national, or regional DRL value. Median doses are an additional tool for dose optimization activities. However, if local practices result in radiation doses far below median dose values, adequate image quality should be ensured [17].

Previously published DRLs for clinical indications were mainly from survey-based methods or from selective submission to established registries. These are likely not as accurate as doses based on a comprehensive assessment such as included in this report. Larger surveys are necessary to account for variations based on size and variations that may result from inclusion of a single or small number of scanners [25]. The recently published EUCLID study is also based on a large-scale survey that collected at least 20 CT examinations of standard-sized patients from each hospital for each EUCLID indication [21]. The differences in the DRLs of the UCSF CT International Dose Registry and this study may be mainly attributable to the inclusion of all patients and all CT scans in the registry rather than only selected patients.

The primary limitation in these data is that assignment to the EUCLID categories may be inaccurate. We assessed the CT doses for the EUCLID clinical indications using data stored within the DICOM data and if these data were non-specific (e.g., the indication was coded only as a routine CT), we would have not been able to correctly assign the CT to the appropriate category. The observation that all of the facilities did not contribute to all EUCLID categories likely reflects our inability to assign some CT scans to the appropriate category, rather than reflecting that the facility did not performing certain types of scans. However, this likely would have added random noise to our estimates and thus diminished differences. Hence, differences between categories may be even greater than reported. Other limitations include the relatively small size of the European studies, the use of only one dose-monitoring software as data source, and that we did not include a measure of imaging quality, as no agreed upon measure exists.

In conclusion, dose metrics from large multi-center studies can help create representative DRLs. These DRLs can be used for dose optimization and institutional evaluation to examine whether institutions routinely exceed these benchmarks. This analysis supports that the clinical indication-specific categories provide more nuanced and accurate reflections of dose requirements that categories based on anatomic area alone. The provided DRLs and median doses for the EUCLID indications can be used as a tool for defining these benchmarks. These benchmarks exceed previously published DRLs for some indications, perhaps providing evidence that practice can be improved, and a greater standardization is needed. Similarly, the higher values for US institutions also might suggest the opportunity for additional dose reduction in the US.

## Supplementary Information

Below is the link to the electronic supplementary material.

Supplementary file1 (DOCX 62.1 KB)

## Abbreviations

AD: Achievable dose
CI: Confidence interval
DRL: Diagnostic reference level
EUCLID: European Study on Clinical Diagnostic Reference Levels for X-ray Medical Imaging

## Declarations

### Guarantor

The scientific guarantor of this publication is Prof. Rebecca Smith-Bindman.

### Statistics and Biometry

One of the authors has significant statistical expertise.

### Methodology

• prospective

• multicenter study
