# Supplementary material for: Diagnostic reference levels and median doses for common clinical indications of CT: findings from an international registry
Source: Eur Radiol. 2021 Oct 13;32(3):1971–82. doi: 10.1007/s00330-021-08266-1 (PMC8831291; doi:10.1007/s00330-021-08266-1)
Supplement: Supplementary file 1 — Supplementary file1 (DOCX 62.1 KB) [file 330_2021_8266_MOESM1_ESM.docx]

**ELECTRONIC SUPPLEMENTARY MATERIAL**

**Supplement 1** Diagnostic reference levels (DRLs) and median doses for CTDI_vol_ and DLP by EUCLID category adjusted for patient size and age.

| **Body Region** | **EUCLID category** | **CTDI_vol_ (mGy)** | | **DLP (mGy⋅cm)** | |
| --- | --- | --- | --- | --- | --- |
|  |  | Median | DRL (75^th^) | Median | DRL (75^th^) |
|  |  |  |  |  |  |
| Head | Chronic sinusitis | 17.6 | 25.1 | 291 | 428 |
|  | Stroke | 47.6 | 55.3 | 876 | 1,058 |
|  |  |  |  |  |  |
| Neck | Cervical spine trauma | 17.8 | 23.3 | 414 | 604 |
|  |  |  |  |  |  |
| Chest | Coronary calcium scoring | 5.7 | 7.8 | 89 | 122 |
|  | Lung cancer | 8.1 | 11.4 | 311 | 459 |
|  | Pulmonary embolism | 10.6 | 14.7 | 403 | 581 |
|  | Coronary CT angiography | 13.4 | 25.5 | 428 | 880 |
|  |  |  |  |  |  |
| Abdomen | Hepatocellular carcinoma | 9.6 | 12.3 | 1,337 | 1,735 |
|  | Colic/Abdominal pain | 9.8 | 12.5 | 507 | 640 |
|  | Appendicitis | 11.5 | 14.5 | 643 | 895 |
|  |  |  |  |  |  |

**Supplement 2** Published diagnostic reference levels (DRLs) and observed EUCLID DRLs based on registry data for CTDI_vol_ and DLP

| **Indication for CT** | **DRLs for CTDI_vol_ (mGy)** | **DRLs for DLP (mGy**⋅**cm)** |
| --- | --- | --- |
| **Chronic sinusitis** | | |
| UCSF CT International Dose Registry 2019 | 24 | 399 |
| Agence fédérale de Contrôle nucléaire (AFCN) (BE) 2018 [26] | 6 | 80 |
| Wachabauer et al. (AT) 2017 [27] | - | 90 |
| German Federal Office for Radiation Protection (DE) 2016 [28] | 8 | 90 |
| Aberle et al. (CH) 2020 [29] | 6 | 90 |
| Schegerer et al. (DE) 2017 [30] | 9 | 120 |
| van der Molen et al. (NL) 2013 [31] | - | 133 |
| Radiation and Nuclear Safety Authority (FI) 2013 [32] | 13 | 190 |
| Foley et al. (IE) 2012 [33] | 16 | 206 |
| **Stroke** | | |
| UCSF CT International Dose Registry 2019 | 55 | 1076 |
| Radiation and Nuclear Safety Authority (FI) 2013[32] | 55 | 800 |
| German Federal Office for Radiation Protection (DE) 2016 [28] | 60 | 850 |
| Aberle et al. (CH) 2020 [29] | 51 | 890 |
| Agence fédérale de Contrôle nucléaire (AFCN) (BE) 2018 [26] | 50 | 900 |
| van der Molen et al. (NL) 2013 [31] | - | 936 |
| Kanal et al. (USA) 2017 (14-16 cm in size) [34] | 56 | 962 |
| Public Health England (UK) 2016 [35] | 60 | 970 |
| Wallace et al. (AU) 2015 [36] | 61 | 992 |
| Wachabauer et al. (AT) 2017 [27] | - | 1100 |
| Japan Network for Research and Information on medical Exposure (JP) 2015 [37] | 85 | 1350 |
| Salama et al. (EG) 2017 [38] | 30 | 1360 |
| Palorini et al. (IT) 2014 (total DLP) [39] | 69 | 1382 |
| **Cervical spine trauma** | | |
| UCSF CT International Dose Registry 2019 | 23 | 589 |
| German Federal Office for Radiation Protection (DE) 2016 [28] | 20 | 300 |
| van der Molen et al. (NL) 2013 [31] | - | 321 |
| Aberle et al. (CH) 2020 [29] | 17 | 360 |
| Foley et al. (IE) 2012 [33] | 19 | 418 |
| Public Health England (UK) 2018 - Holroyed et al. 2018 [40] | 20 | 440 |
| Agence fédérale de Contrôle nucléaire (AFCN) (BE) 2018 [26] | 25 | 450 |
| Kanal et al. (USA) 2017 (17-21 cm in size) [34] | 28 | 562 |
| Wallace et al. (AU) 2015 (neck/cervical spine) [36] | 32 | 597 |
| Public Health England (UK) 2016 [35] | 28 | 600 |
|  | | |
| **Coronary calcium scoring** | | |
| UCSF CT International Dose Registry 2019 | 7 | 106 |
| van der Molen et al. (NL) 2013 [31] | - | 51 |
| Schegerer et al. (DE) 2017 [30] | 8 | 119 |
| Palorini et al. (IT) 2014 [39] | 7 | 131 |
| Treier et al. (CH) 2010 [41] | 10 | 150 |
| **Lung cancer** | | |
| UCSF CT International Dose Registry 2019 | 13 | 524 |
| Aberle et al. (CH) 2020 [29] | 7 | 250 |
| Radiation and Nuclear Safety Authority (FI) 2013 [32] | 11 | 430 |
| Wachabauer et al. (AT) 2017 (per scan) [27] | - | 500 |
| Public Health England (UK) 2016 [35] | 12 | 610 |
| Danish Health Authority (DK) 2015 [42] | 16 | 620 |
| **Pulmonary embolism** | | |
| UCSF CT International Dose Registry 2019 | 16 | 631 |
| Schegerer et al. (DE) 2017 [30] | 15 | 300 |
| Aberle et al. (CH) 2020 [29] | 8 | 300 |
| van der Molen et al (NL) 2013 [31] | - | 371 |
| Foley et al. (IE) 2012 [33] | 13 | 432 |
| Public Health England (UK) 2016 [35] | 13 | 440 |
| Kanal et al. (US) 2017 (29-33 cm in size) [34] | 14 | 445 |
| **Coronary CT angiography** | | |
| UCSF CT International Dose Registry 2019 | 25 | 935 |
| Danish Health Authority (DK) 2015 [42] | 29 | 230 |
| Castellano et al. (UK) 2017 [43] | - | 336 |
| German Federal Office for Radiation Protection (DE) 2016 [28] | 20 | 330 |
| Mafalanka et al. (FR) 2015 (prospective ECG-gating mode) [44] | 26 | 370 |
| Agence fédérale de Contrôle nucléaire (AFCN) (BE) 2018 (total DLP) [26] | 25 | 480 |
| van der Molen et al. (NL) 2013 [31] | - | 671 |
| Mafalanka et al. (FR) 2015 (retrospective ECG-gating mode) [44] | 44 | 870 |
| Treier et al. (CH) 2010 [41] | 50 | 1000 |
| Hausleiter et al. 2009 [45] | 69.6 | 1152 |
| Palorini et al. (IT) 2014 [39] | 61 | 1208 |
| Japan Network for Research and Information on medical Exposure (JP) 2015 [37] | 90 | 1400 |
| Fukushima et al. (JP) 2012 [46] | - | 1510 |
| **Hepatocellular carcinoma** | | |
| UCSF CT International Dose Registry 2019 | 15 | 2032 |
| Aberle et al. (CH) 2020 [29] | 11 | 1170 |
| Salama et al. (EG) 2017 (liver metastasis, three phases) [38] | 31 | 1425 |
| **Colic/Abdominal pain** | | |
| UCSF CT International Dose Registry 2019 | 15 | 817 |
| Aberle et al. (CH) 2020 [29] | 6 | 280 |
| van der Molen et al. (NL) 2013 [31] | - | 329 |
| Radiation and Nuclear Safety Authority (FI) 2013 [32] | 7 | 330 |
| Public Health England (UK) 2016 [35] | 10 | 460 |
| **Appendicitis** | | |
| UCSF CT International Dose Registry 2019 | 17 | 1044 |
| Aberle et al. (CH) 2020 (Abdomen-pelvis) [29] | 11 | 540 |
| Foley et al. (IE) 2012 (Routine abdomen) [33] | 12 | 598 |
| Wachabauer et al. (AT) 2017 (Acute abdomen, per scan) [27] | - | 650 |
| Danish Health Authority (DK) 2015 (Acute abdomen) [42] | 17 | 700 |
| Public Health England (UK) 2016 (Abscess) [35] | 15 | 745 |
|  |  |  |
